# Dirichlet Diffusion Score Model for Biological Sequence Generation

**Published:** 2023-05-18

**Authors:** Pavel Avdeyev, Chenlai Shi, Yuhao Tan, Kseniia Dudnyk, Jian Zhou

**Affiliations:** 1 Lyda Hill Department of Bioinformatics, University of Texas Southwestern Medical Center, USA.

## Abstract

Designing biological sequences is an important challenge that requires satisfying complex constraints and thus is a natural problem to address with deep generative modeling. Diffusion generative models have achieved considerable success in many applications. Score-based generative stochastic differential equations (SDE) model is a continuous-time diffusion model framework that enjoys many benefits, but the originally proposed SDEs are not naturally designed for modeling discrete data. To develop generative SDE models for discrete data such as biological sequences, here we introduce a diffusion process defined in the probability simplex space with stationary distribution being the Dirichlet distribution. This makes diffusion in continuous space natural for modeling discrete data. We refer to this approach as Dirchlet diffusion score model. We demonstrate that this technique can generate samples that satisfy hard constraints using a Sudoku generation task. This generative model can also solve Sudoku, including hard puzzles, without additional training. Finally, we applied this approach to develop the first human promoter DNA sequence design model and showed that designed sequences share similar properties with natural promoter sequences.

## 1. Introduction

Diffusion probabilistic models are a family of models that reverse diffusion process to generate data from noise. Score-based generative stochastic differential equation (SDE) is a type of continuous-time diffusion model that has many desirable properties, such as allowing likelihood evaluation through a connection to a probability flow ordinary differential equation (ODE) and flexibility in sampling approaches. However, the originally proposed generative SDEs are not directly suitable for modeling discrete data. Recent works have proposed methods for adapting diffusion models to discrete data (Campbell et al., 2022; Chen et al., 2022b; Sun et al., 2022; Austin et al., 2021; Hoogeboom et al., 2021b;a), including continuous-time diffusion in discrete space (Campbell et al., 2022), but no methods are formulated within the continuous-time SDE diffusion framework (Song et al., 2020), except for quantization-based methods. In this manuscript, we propose a general mechanism to extend this approach to discrete data, while allowing continuous-time diffusion in probability simplex space. Specifically, designed to utilize the natural connection between Dirichlet distribution and discrete data, we consider continuous-time diffusion within the probability simplex for which the stationary distribution is Dirichlet distribution. Forward diffusion (data-to-noise) of discrete data starts from the vertices of the probability simplex space, and diffuses continuously in the same space, and the continuous-discrete space gap can be bridged with a latent variable interpretation.

While our intended application is in biological sequence generation, we evaluated our, Dirichlet diffusion score model (DDSM)^1^, on a range of discrete data generation tasks to better understand its performance. In addition to demonstrating competitive performance on a small benchmark dataset, binarized MNIST, we applied it to generating Sudoku puzzles to test for its ability in generating highly structured data with strong constraints. The model can not only generate but also solve Sudoku puzzles including hard puzzles, which is the first time this is achieved with a purely generative modeling approach. Finally, we applied DDSM to a real-world application in biological sequence generation. Specifically, we developed the first model for designing human promoter DNA sequences that drive gene expression, and demonstrate that it designs diverse sequences comparable to human genome promoter sequences.

## 2. Background

### 2.1. Score-based Generative Modeling with SDE

Itô diffusion process is defined as

$$dx=f\left(x,t\right)dt+G\left(x,t\right)dw,$$

where w is the standard Wiener process (a.k.a., Brownian motion), the drift coefficient $f(\cdot ,t):R^{n}\to R^{n}$ and diffusion coefficient $G(\cdot ,t):R^{n}\to R^{n}$ are vector-valued functions of $x^{t}$. Song et al. 2020 exploited the remarkable result by Anderson 1982 that the time-reversal of this diffusion process can be obtained by the following SDE:

(1) $$\begin{array}{l} dx=\{f(x,t)-\nabla \cdot [G(x,t)G(x,t{)}^{⊤}]\\ \left.-G(x,t)G(x,t{)}^{⊤}\nabla_{x}logp_{t}(x)\right\}dt\\ \;+G(x,t)d\overset{-}{w}, \end{array}$$

where $\nabla \cdot [G(x,t)G(x,t{)}^{⊤}]$ indicates row-sums of element-wise derivative with respect to x. The corresponding probability flow ODE is defined as

(2) $$\begin{array}{l} dx=\{f(x,t)-\frac{1}{2}\nabla \cdot [G(x,t)G(x,t{)}^{⊤}]\\ \;-\frac{1}{2}G(x,t)G(x,t{)}^{⊤}\nabla_{x}logp_{t}(x)\}dt. \end{array}$$

It gives the same distribution at time t as the reverse-time SDE. Both the reverse-time SDE and the probability flow ODE can be sampled from given $\nabla_{x}logp_{t}(x)$ or the score of $p_{t}(x)$. Therefore, learning the reverse diffusion becomes the problem of learning the score function, which is usually parameterized as a neural network known as the *score model*. The training loss is the score matching loss

(3) $$\int_{0}^{T}\;E_{p_{t}\left(x^{t}\right)}\left[\lambda (t){∥\nabla_{x}logp\left(x^{t}\right)-s_{\theta }\left(x^{t},t\right)∥}_{2}^{2}\right]dt,$$

where $s_{\theta }(x,t)$ is the score model to be trained, and λ(t) is a positive weighting function. The loss is equivalent to the denoising score matching loss

(4) $$\begin{array}{rr} \int_{0}^{T}\;\;E_{p_{0}\left(x^{0}\right)p\left(x^{t}|x^{0}\right)}\left[\lambda (t)∥\nabla_{x}\right. & logp\left(x^{t}|x^{0}\right)\\ \left.-s_{\theta }\left(x^{t},t\right){∥}_{2}^{2}\right]dt, \end{array}$$

which is used in practice because $\nabla_{x}logp\left(x^{t}|x^{0}\right)$ is usually easier to compute. Forward diffusion processes considered so far have Gaussian stationary distribution and are applicable for continuous data in $R^{n}$.

### 2.2. Univariate Jacobi Diffusion Process

We consider Jacobi diffusion process^2^ in the following form

(5) $$dx=\frac{s}{2}\left[a\left(1-x\right)-bx\right]dt+\sqrt{sx\left(1-x\right)}dw,$$

where 0≤x≤1,s>0 is the speed factor, and a>0,b>0 determines the stationary distribution **Beta** (a,b). We usually use s=1 or $s=\frac{2}{a+b}$ (see Appendix A.2 for discussion of choices). Note that when x approaches 0 or 1, the diffusion coefficient converges to 0 and the drift coefficient converges to a or −b, keeping the diffusion within [0,1].

The spectral expansion of the transition density function was derived by Kimura 1955; 1957. Hence, the diffused density at any time t is computed by the following formula:

(6) $$\begin{array}{l} p_{a,b}\left(x^{t}|x^{0}\right)\\ ={ℬ}_{a,b}\left(x^{t}\right)\sum_{n=0}^{\infty }\frac{e^{\lambda_{n}t}}{d_{n}}R_{n}^{\left(a,b\right)}\left(x^{0}\right)R_{n}^{\left(a,b\right)}\left(x^{t}\right)\\ ={ℬ}_{a,b}\left(x^{t}\right)\left(1+\sum_{n=1}^{\infty }\frac{e^{\lambda_{n}t}}{d_{n}}R_{n}^{\left(a,b\right)}\left(x^{0}\right)R_{n}^{\left(a,b\right)}\left(x^{t}\right)\right), \end{array}$$

where ${ℬ}_{a,b}\left(x_{t}\right)$ is the Beta(a,b) density, $R_{n}^{\left(a,b\right)}(x)$ denotes the n-th order modified Jacobi polynomial of order n and are eigenfunctions of the generator of the Jacobi diffusion process (Steinrücken et al., 2013a; Griffiths & Spano’, 2010). The corresponding eigenvalues are $\lambda_{n}=-\frac{1}{2}sn(n-1+a+b)$. The gradient of the log transition density function can be computed via automatic differentiation.

## 3. Diffusion Processes for Generative SDE Modeling of Discrete Data

### 3.1. Forward Diffusion SDE for Two-Category Data

**Figure 1.**
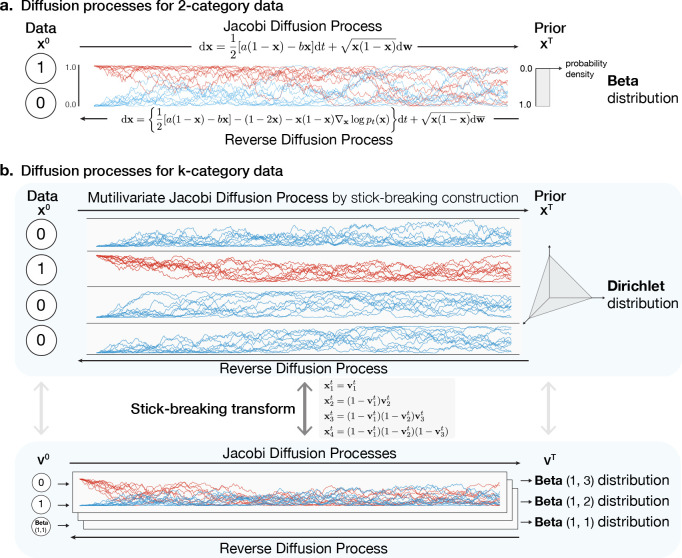
Schematic overview of forward and reverse diffusion SDEs for Dirichlet diffusion score model. Forward and reverse diffusion SDEs for 2-category data (a) and k-category data (b) by stick-breaking construction are shown.

Using the univariate Jacobi diffusion as the forward diffusion process provides a natural generalization of the score-based generative SDE approach (Section 2.1) to discrete data with two categories, encoded as 0 and 1. The forward diffusion starting from 0 or 1 at the initial timepoint will continuously diffuse in the [0,1] interval and converge to a Beta stationary distribution (see Fig. 1a). If a=1 and b=1 in Eq. 5 and 6 then the Beta stationary distribution is **Beta**(1,1) (i.e., a uniform distribution in the interval [0,1]).

The score-based generative SDE model can be trained via the denoising score matching objective (see Eq. 4) following the transition density formula (see Eq. 6). By combining equations 1 and 5, we have the following reverse-time SDE:

(7) $$\begin{array}{l} \text{d}x=\{\frac{s}{2}[a(1-x)-bx]-s(1-2x)\\ \;-sx(1-x)\nabla_{x}\log p_{t}(x)\}\text{d}t\\ \;+\sqrt{sx(1-x)}\text{d}\bar{w}. \end{array}$$

Replacing $\nabla_{x}logp_{t}(x)$ with score model $s_{\theta }\left(x^{t},t\right)$ allows sampling from the trained model via reverse diffusion.

### 3.2. Forward Diffusion SDE for k-Category Data

To model discrete variables with k categories, e.g. DNA sequence (with four bases A,C,G,T) or protein sequence (20 amino acid residues), we need to consider diffusion in probability simplex.

We seek to use a multivariate diffusion process over the probability simplex for which the stationary distribution is Dirichlet distribution (see Fig. 1b). Jacobi diffusion process converges to Beta stationary distribution, a univariate special case of Dirichlet distribution. Using the connection between Beta distribution and Dirichlet distribution, we construct a multivariate diffusion process on probability simplex that converges to Dirichlet distribution with k−1 independent univariate Jacobi diffusion processes by a classical stick-breaking construction

$$x_{1}^{t}=v_{1}^{t},x_{2}^{t}=\left(1-v_{1}^{t}\right)v_{2}^{t},x_{3}^{t}=\left(1-v_{1}^{t}\right)\left(1-v_{2}^{t}\right)v_{3}^{t},\cdots ,$$

where $v_{1}^{t},v_{2}^{t},\ldots ,v_{k-1}^{t}$ are drawn from independent Jacobi diffusion processes at time t. Thus, we obtain a multivariate diffusion process with Dirichlet stationary distribution using $x_{1}^{t},x_{2}^{t},\ldots ,x_{k}^{t}$.

For notation simplicity, we will use v and x to indicate the k−1 and k dimensional representations for the rest of the manuscript, respectively. The conversion between v and x is done by stick-breaking transform and its inverse.

For obtaining any Dirichlet stationary distribution, we parameterize the Jacobi diffusion process. For example, for the stationary distribution to be the flat Dirichlet distribution Dir⁡(1,1,…,1) (i.e., the uniform distribution over the probability simplex), we need to choose the Jacbobi diffusion processes with stationary distributions Beta⁡(1,k−1),Beta⁡(1,k−2),…,Beta⁡(1,1) for $v_{1},v_{2},\ldots ,v_{k-1}$ of stick-breaking construction. This can be simply achieved by choosing parameters a, b in equation 5 to be (1,k−1),(1,k−2),…,(1,1).

We refer to this multivariate diffusion process as *multivariate Jacobi diffusion process by stick-breaking construction,* and the generative modeling approach with this diffusion process as *Dirichlet diffusion score model.* An infinite dimensional form of this diffusion process with a more general distribution family has been proposed as the GEM process (Feng & Wang, 2007). The proposed process is a finite-dimensional version of the GEM process. We note that other forms of diffusion processes for which the stationary distribution is Dirichlet distribution exist (see Steinrücken et al. 2013b; Bakosi & Ristorcelli 2013). However, they are much more computationally expensive to use as forward diffusion processes since they cannot be decomposed into independent univariate diffusion processes.

### 3.3. Score Matching Training for k-Category Discrete Data

Using the multivariate Jacobi diffusion by stick-breaking construction as the forward diffusion process allows us to train score-based diffusion model for k-category discrete data.

The initial value of the diffusion in x space is set to be the discrete data represented by k-dimensional one-hot encoding such as (0,0,1,…,0). To sample from the forward diffusion, we first map the initial values of x to v space via inverse stick-breaking transform. For all dimensions of v after the first 1 that is undetermined by inverse stick-breaking transform given x, we consider them as drawn from corresponding stationary Beta distribution. Explicitly drawing the Beta samples for initial values are not needed since the density remains stationary and the samples and scores at any time are directly computed from the Beta distributions.

The forward diffusion samples in v space at any time t are drawn from the Jacobi diffusion processes (for dimension with deterministic initial values) and stationary Beta distributions (for dimension with undetermined initial values). The scores for the transition density function in denoising score-matching loss (Eq. 4) are then computed from the corresponding Jacobi diffusion transition density function and Beta density function.

By applying the change-of-variable conversion, we can equivalently perform score matching in either v space or x space since we can convert between the score of x to the score of v:

$$\frac{\partial \log p_{x}(x)}{\partial x}=\left(\frac{\partial \log p_{v}(v)}{\partial v}+\frac{\partial \log\left|\det\frac{\partial v}{\partial x}\right|}{\partial v}\right)\frac{\partial v}{\partial x},$$

$$\frac{\partial \log p_{v}(v)}{\partial v}=\frac{\partial \log p_{x}(x)}{\partial x}\frac{\partial x}{\partial v}-\frac{\partial \log\left|\det\frac{\partial v}{\partial x}\right|}{\partial v}.$$

The score model $s_{\theta }\left(x^{t},t\right)$ is more naturally formulated as a function of x, and we choose to compute score-matching loss in v space because of the diagonal form of diffusion coefficient.

Once the score model is learned, sampling from the reverse diffusion process in v space is nearly identical to sampling from multiple univariate reverse diffusion processes as in Equation 7, except for that the score model takes all dimensions of v as input (after converting to x space).

### 3.4. Weighting Function for Score Matching Loss of General SDE

The choice of weighting function λ(t) in score matching loss (Eq. 3 and 4) has previously been studied (Song et al., 2021; Huang et al., 2021). With the assumption that the scalar diffusion coefficient g(v,t) of the forward diffusion process does not depend on the value v being diffused, $\lambda (t)=g(t{)}^{2}$ is shown to be the likelihood weighting (Song et al., 2021). Minimizing the loss function with this weighting is equivalent to maximizing the ELBO (Huang et al., 2021). However, this assumption about g(v,t) does not hold for the Jacobi diffusion process.

Here we motivate the use of

(8) $$L(v,t)={\left\|\frac{\partial \log p_{v}(v)}{\partial v}-\frac{\partial \log q_{v}(v)}{\partial v}\right\|}_{{\text{GG}}^{T}}^{2}\;\;=\left(\frac{\partial \log p_{v}(v)}{\partial v}-\frac{\partial \log q_{v}(v)}{\partial v}\right)G(v,t)\;\;{\;G(v,t)}^{T}{\left(\frac{\partial \log p_{v}(v)}{\partial v}-\frac{\partial \log q_{v}(v)}{\partial v}\right)}^{T}$$

to be the general form of weighted score-matching loss for any SDE with matrix-form diffusion coefficient G(v,t), from the argument that the loss function should satisfy the property of invariance under change-of-variable, which is satisfied by likelihood function. In Appendix A.3, we show that this loss function is invariant to change-of-variable by any bijective, differentiable function x=h(v), while the unweighted loss is not. If G is scalar or diagonal and does not depend on v, we recover the likelihood weighting from Song et al. 2020.

### 3.5. Likelihood Computation

After the model is trained with score-matching, we can estimate likelihood using probability flow ODE (see Eq. 2). Our formulation allows both computing the likelihood from the continuous distribution over the probability simplex, and computing a variation lower-bound of the likelihood of discrete data.

#### LIKELIHOOD COMPUTATION FOR CONTINUOUS VARIABLE IN PROBABILITY SIMPLEX

We will first estimate the likelihood in the v space, which can be easily converted to likelihood in the x space using the probability change-of-variable formula (Chen et al., 2018).

Following Song et al. 2021, the probability flow ODE for a Jacobi diffusion process is:

$$dv=\{\frac{s}{2}\left[a\left(1-v\right)-bv\right]\;\;-\frac{s}{2}\left(1-2v\right)-\frac{s}{2}v\left(1-v\right)s_{\theta }\left(v,t\right)\}dt=\tilde{f}dt$$

By the instantaneous change-of-variable formula, we have:

$$p_{0}\left(v^{0}\right)=e^{\int_{0}^{t}\;\;tr\left(\nabla_{v}\tilde{f}\left(v^{t}\right)\right)dt}p_{t}\left(v^{t}\right)$$

The trace of Jacobian can be unbiasedly approximated by Hutchinson’s estimator

$$tr\left(\nabla_{v}\tilde{f}\left(v^{t}\right)\right)=E_{\epsilon \;\sim 𝒩(0,I)}\left[\epsilon^{T}\nabla_{v}\tilde{f}\left(v^{t}\right)\epsilon \right]$$

and $p_{t}\left(v^{t}\right)$ is computed with the stationary distribution. $p_{0}\left(v^{0}\right)$ is converted to $p_{0}\left(x^{0}\right)$ by applying the change-of-variable formula to obtain the likelihood.

Since this continuous-space likelihood is not directly comparable with discrete-space likelihood, next we will derive an evidence lower bound (ELBO) that allows direct comparison with likelihood in discrete data space.

#### BOUNDING DISCRETE DATA LIKELIHOOD WITH VARIATIONAL LOWERBOUND

To obtain a variational lowerbound for discrete likelihood, we consider the continuous variable x in probability simplex space, drawn from the reverse diffusion process, as directly parameterizing categorical distributions. The discrete data y are drawn from these categorical distributions. Thus we obtain the discrete likelihood by marginalizing over $xp(y)=\int p^{Cat}(y|x)p(x)dx$.

While this is generally intractable computationally, we use the variational lowerbound ELBO

$$\begin{array}{r} logp(y)\geq E_{q^{Diff}(x|y)}\left[-logq^{Diff}(x|y)\right.\;+logp^{Cat}(y|x)\\ \left.+logp^{ODE}(x)\right], \end{array}$$

where $q^{\text{Diff}}(x|y)$ is the density of forward diffusion from y at time $t_{\tilde{0}}$, with $t_{\tilde{0}}$ chosen to be close to $0.p^{Cat}(y|x)$ is the categorical distribution likelihood. $p^{ODE}(x)$ is the continuous-space likelihood of probability flow ODE as described in the previous subsection, but with the lower end of time being $t_{\tilde{0}}$ instead of 0. This expectation is unbiasedly estimated by sampling from the forward diffusion process. This ELBO formulation is chosen so that the diffusion model training will also minimize the variational gap of this ELBO, which reduces to the KL divergence between the forward diffusion density and the reverse diffusion density up to a constant when $t_{\tilde{0}}\to 0$. This bound can be tightened by choosing $t_{\tilde{0}}$ closer to zero. This bound is fairly tight in practice when $t_{\tilde{0}}$ is small, and we performed an empirical analysis on a simple test case (see Appendix B.8).

### 3.6. Improving Sampling Efficiency and Sample Quality

Lastly, we introduce two techniques that can be applied to improve the efficiency of forward diffusion sampling during training, or improve sample quality post-training, which are both detailed in Appendix. The sampling strategy for the forward diffusion process presented in Section 3.3 requires drawing samples from k−1 Jacobi diffusion processes for k-category data, which can be demanding when k is high. In Appendix A.4, we describe a strategy to simplify sampling, needing to effectively sample from only one univariate Jacobi diffusion process.

The second technique is designed to improve sample quality. Comparing to unbiasedly sampling from the learned model distribution, it is often desirable to sample near the high probability density regions, which often corresponds to higher quality samples in suitable applications. In Appendix A.5 we propose a simple technique, *time-dilation*, applicable to reverse diffusion sampling without modifying the score model, when a flat distribution such as the flat Dirichlet distribution is the stationary distribution. In Appendix B.9, we compare sample quality obtained by time dilation with other sampling strategies which reported to improve sample quality (Song et al., 2020).

## 4. Results

### 4.1. Implementation Notes of Dirichlet Diffusion Score Model

Sampling from Jacobi diffusion processes is more expensive than commonly used SDEs with Gaussian stationary distributions (Song et al., 2020), as we need an SDE sampler such as Euler-Maruyama sampler. However, we only need to generate samples from two starting points, 0 and 1, for any categorical data. Hence, we can pre-sample a dictionary of diffused samples at different time points t and sample from the dictionary during training time. Similarly, the log transition density function gradient at the samples can also be precomputed. This approach allows efficient training with little additional overhead. We discuss additional implementation details in the Appendix B.

### 4.2. Application to Binarized MNIST

We first applied the method to a benchmark dataset for generative modeling, the binarized MNIST dataset, and obtained competitive performance (Table 1). More details of all applications are included in Appendix C. Examples of samples are shown in Appendix D.

### 4.3. Sudoku Generation as a Constraint Satisfying Generation Test

To test the ability to generate highly structured data that satisfy hard constraints, we applied our method to the problem of generating and solving Sudoku. This problem has not been solved through generative modeling to the best of our knowledge.

For training, we used a Sudoku generator to continuously produce Sudoku puzzles. A fully-filled Sudoku puzzle can be encoded with 81 categorical variables with the number of categories k=9. The model architecture we adopt is based on a 20-block transformer architecture with a relative positional encoding designed for the Sudoku problem.

Specifically, a Sudoku puzzle is represented by a set of 81 elements and a binary relative positional encoding that is 27 dimensional (81×81×27), corresponding to whether two elements are of the same row, same column, or the same 3×3 block. The relative positional encoding is transformed by a linear layer and added to the transformer’s attention prior to the softmax.

The generative capability of the model is evaluated by the percentage of generated Sudoku that is correctly filled (Table 2). Only whether a Sudoku is completely correct is considered, with no partial credit given. Applying the time-dilation technique (Appendix B.3) to drive samples toward high-density areas improved sample quality to up to 100%. In contrast, the heuristic algorithm for generating of Sudoku that we used to generate training data, has only 0.31% success rate. Even though no previous generative modeling approach has been applied to Sudoku, we also trained the Sudoku Transformer with Bit Diffusion (Chen et al., 2022b) and D3PM-uniform/Multinomial Diffusion (Hoogeboom et al., 2021b; Austin et al., 2021), using the same model architectures. DDSM with time dilation achieved the best performance in comparison with these methods (Appendix C.3).

Interestingly, similar to prior observations on image generation quality (Song et al., 2020), we also observe a trade-off between Sudoku-solving ability and computational budget, with improved Sudoku-generation accuracy using a higher number of sampling steps and time-dilation.

### 4.4. Solving Sudoku via Conditional Generation

We applied the Sudoku generative SDE model to solving Sudoku puzzles by a conditional generation with the inpainting method (Song et al., 2020) of clamping entries to the given clues of the puzzle. We evaluated the model on an easy Sudoku dataset with 36 clues on average (Wang et al., 2019) and a hard Sudoku dataset with minimally 17 clues (Palm et al., 2018), which is the minimum number of clues possible for Sudoku (McGuire et al., 2014).

Even though no additional training is done for solving Sudoku, the generative SDE model trained with DDSM solved most puzzles in the easy dataset with a single sample (Table 2). In contrast, both models trained with Bit Diffusion and D3PM-uniform have difficulties in the Sudoku solving task, with only <10% of easy puzzles solved with a single sample.

The Sudoku-solving performance of the DDSM model can be further improved by increasing time-dilation, and a single sample solves 99.4% of the easy dataset and 42.4% of the hard dataset (128x time-dilation). When multiple samples are allowed, the model can solve 100% of all puzzles. The number of samples required to solve a Sudoku puzzle significantly increases with lower than 25 clues, with about 2.3x increase per one fewer clue given which is still significantly better than random guesses (Appendix E). This allows us to solve 100% hard Sudoku puzzles in the dataset. Neither models trained with Bit Diffusion nor D3PM-uniform have the ability to solve hard puzzles. Previous state-of-the-art supervised models SATNET (Wang et al., 2019) and Recurrent relational network (Palm et al., 2018) can solve most but not all of them (see Appendix E for more discussion).

### 4.5. Generation of Promoter DNA Sequences

**Figure 3.**
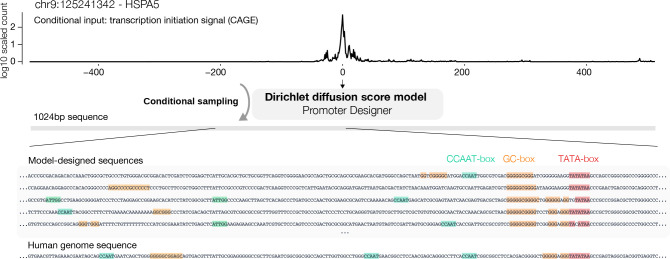
Design human gene promoter sequences with conditional Dirichlet diffusion score model trained to generate sequences from transcriptional initiation signal profile. Example model-designed sequences based on a transcriptional initiation signal profile of a test set promoter are shown. The corresponding human genome sequence is shown in comparison. Known promoter motifs are annotated in the sequences. We provide an introduction for readers unfamiliar with this topic in Appendix F.1.

Finally, we applied the method to designing human promoter DNA sequences (Figure 3). Promoters are key DNA sequences that drive the transcription of genes and partially determine gene expression levels. Designing promoter sequences can have broad applications in biomedical research and bioengineering applications, such as controlling synthetic gene expression. Human promoter sequences are known to be highly diverse and rules that determine promoter sequence activity are not fully understood (Wang et al., 2017). Thus it is an ideal problem to be addressed through deep generative modeling. No prior computational approach for designing human promoter sequences exists to our knowledge.

To enable human promoter sequence design, we trained a conditional Dirichlet diffusion score model to perform conditional generation of promoter sequences using transcription initiation signal profile as an additional input to the score model (Figure 3). Transcription initiation signal profiles reflect the transcription initiation activity at every sequence position and are obtained from CAGE experiments (Consortium et al. 2014). The conditional generation model allows controlling the transcription initiation signal profile produced by the sequence, including controlling the expression level. We constructed the human promoter sequence dataset containing 100,000 promoter sequences and corresponding transcription initiation signal profiles, with each sequence 1024 basepairs long and centered at the annotated transcription start site position (Hon et al., 2017). This set of promoters spans the whole range of human promoter activity levels from highly expressed protein-coding gene promoters to ncRNA gene promoters with very low expression.

### 4.6. Evaluation of Designed Promoter DNA Sequences

**Figure 4.**
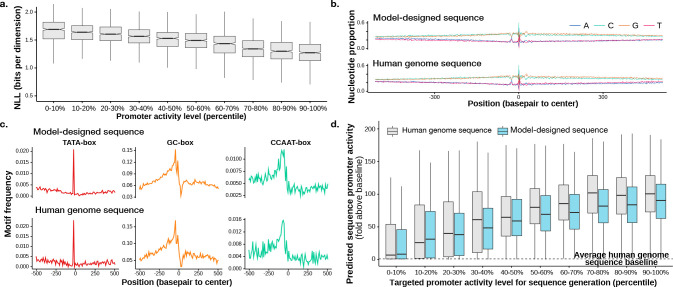
Performance of promoter design diffusion sequences model. a) NLL evaluated on test chromosomes 8 and 9, grouped by the expression level of the promoter (90–100% percentile being most expressed). (b-c) Model-designed sequences have comparable position-specific nucleotide composition (b) and motif location distribution (c). d) Designed sequences (blue) are compared with human genome sequences (gray) using promoter activity predicted from sequence by Sei (Chen et al., 2022a). Generated sequences are grouped by the targeted promoter activity level (x-axis). Y-axis shows predicted promoter activity (average H3K4me3 prediction across cell types), divided by baseline prediction for average genomic sequences.

With a custom score-model architecture that we call Promoter Designer, the generative model obtained a conditional NLL estimate of ≤ 1.32 bits per basepair for promoters that are from test set chromosomes and among the top 10,000 promoters, whereas simple baselines using position-specific base composition achieves only 1.92 bits. Promoter sequences with higher activity levels also obtained better NLL estimates (Figure 4a), in line with the expectation that sequences of high-activity promoters are less random compared to low-activity promoters. Multiple sequence samples conditioned on the same transcriptional initiation signal profile are typically diverse (Figure 3) while sharing similar characteristics. The generated sequences return no hits when compared with the human genome using BLAST (Morgulis et al., 2008), thus the model does not simply memorize human genomic sequences.

The sequence samples are observed to contain highly similar properties as promoter sequences from the human genome (Figure 4b–d), such as position-specific nucleotide composition relative to the transcription start site (Figure 4b) as well as distribution of known promoter related motifs (Figure 4c)

To evaluate whether the generated sequences recapitulated more complex sequence rules of promoter activity, we applied a published deep learning sequence model Sei that can predict active promoter from sequence (based on chromatin mark H3K4me3 predictions) (Chen et al., 2022a), the generated sequence has comparable predicted promoter activity with the human genome sequence, for both high and low activity promoters (Figure 4d). Applying time-dilation further increased predicted promoter activity (Appendix F).

We also trained models with baseline discrete data diffusion approaches. The evaluation is based on comparing generated sequences and human genome promoter sequences (ground truth) on the test chromosomes similar to Figure 4c. The metric SP-MSE is the MSE between the predicted promoter activity of generated sequences and human genome sequences (lower is better). Our model trained with DDSM outperforms models trained with baseline approaches (Table 3).

## 5. Related Work

Diffusion models were first proposed in Sohl-Dickstein et al. 2015; Ho et al. 2020, including their first application to discrete data using a binomial diffusion process for a binary dataset (Sohl-Dickstein et al., 2015). Song et al. 2020 proposed the score-based generative SDE diffusion model framework with continuous time for continuous data. Recent works for generalizing diffusion models to discrete data have mostly considered discrete time (Hoogeboom et al., 2021b; Austin et al., 2021). More recent works (Campbell et al., 2022; Sun et al., 2022) proposed continuous-time approaches for discrete-space diffusion based on a continuous-time Markov chain formulation. Another direction of work is to apply existing continuous-time continuous-space diffusion approach to discrete data encodings, such as bit encoding (Chen et al., 2022b) or word embedding (Li et al., 2022), and quantize the continuous samples.

Latent diffusion models also allow generative modeling of discrete data by modeling only the distribution of continuous latent variable with diffusion (Vahdat et al., 2021; Lovelace et al., 2022). While the reverse diffusion process in our approach can also be interpreted as a latent variable that emits discrete data (Section 3.5), the relationship between latent and discrete variables is fixed rather than learned.

On generative modeling for biological sequence design, deep generative models have been recently applied to DNA sequence design (Killoran et al., 2017; Wang et al., 2020; Zrimec et al., 2022), even though no diffusion model has been developed. No prior method exists for designing human promoter sequences to our knowledge. On the protein sequence design problem, several deep generative models have been developed to generate sequences conditioned on protein structure (Ingraham et al., 2019; Dauparas et al., 2022), and diffusion models have been applied to generate protein structure (Trippe et al., 2022; Watson et al., 2022; Lee & Kim, 2022). Recently works also jointly generate structure and sequence with diffusion (Luo et al., 2022; Anand & Achim, 2022).

Our contribution is to propose the first approach for discrete data modeling with continuous-time SDE diffusion in probability simplex space, and applied this approach to develop the first method for human promoter sequence design and a novel application to generative modeling of Sudoku. All existing works using score-based generative SDEs are based on diffusion processes that converge to Gaussian stationary distributions, and here we expand the generative SDE toolkit to include ones that converge to Dirichlet stationary distribution. In addition, we propose a simple and easily applicable technique, time-dilation, to improve sample quality.

## 6. Discussion

We provided a continuous-time Dirichlet diffusion score model framework (DDSM), for generative modeling of biological sequences, which can also be used for other types of discrete data. The approach also provides a plug-in substitute for Gaussian stationary distribution SDEs for discrete variables and expands the toolkit of generative diffusion model.

The size of the continuous diffusion state scales linearly with the number of categories and thus may not directly scale to a very high number of categories due to memory and computational constraints. A potential way to address this issue is to use bit encoding or hierarchical encoding schemes that can reduce the encoding dimensions required down to ${log}_{2}(C)$, where C is the number of categories. However, the current approach is ideal for applications in modeling biological sequences, such as DNA and RNA sequences (4 bases) and protein sequences (20 amino acid residues), as well as other data that can be encoded with a moderate number of categories.

We are encouraged by the promising results in designing human promoter sequences and the strong constraint satisfaction capability demonstrated in generating and solving sudoku, and look forward to further development in biological sequence design based on this approach.

## Supplementary Material

Supplement 1

## Figures and Tables

**Figure 2.**
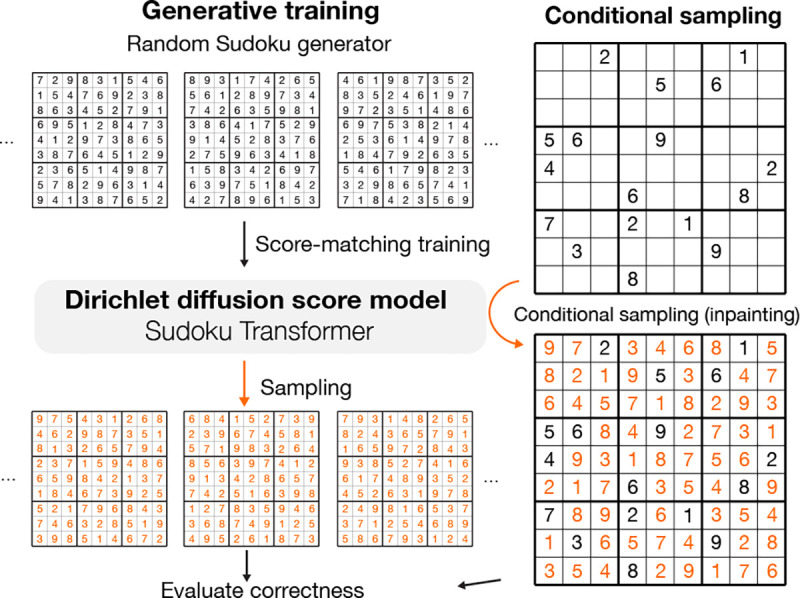
Sudoku generation and solving through generative modeling with diffusion model, as a test for constraint satisfaction.

**Table 1.** Binarized MNIST benchmark performance

Method	NLL (nats) ↓

**DDSM** (ours)	78.04 ± 0.37
CR-VAE	**76.93**
Locally Masked PixelCNN	77.58
PixelRNN	79.20
PixelCNN	81.30
EoNADE	84.68
MADE	86.43
NADE	88.33

**Table 2.** Sudoku generation and solving accuracies for *single samples* with DDSM. With *multiple samples,* all Sudoku puzzles we tested were solved. See Appendix C.3 for comparison with baseline diffusion methods.

Task	Time dilation	Accuracy (%)

Generation	**8x**	**100**
	4x	99.88 ± 0.06
	2x	98.87 ± 0.16
	1x	95.08 ± 0.46
(Heuristic algorithm baseline)		0.31

Solving	**8x**	**98.26** ± **0.18**
	4x	97.54 ± 0.18
	2x	96.45 ± 0.32
	1x	93.85 ± 0.42

**Table 3.** Promoter design performance comparison for different models. We trained all models with the same Promoter Designer architecture and the same early stopping criterion for this comparison.

Model	SP-MSE ↓

**DDSM (time dilation 4x)**	**0.0334**
DDSM (time dilation 2x)	0.0348
DDSM (time dilation 1x)	0.0363
D3PM-uniform / Multinomial Diffusion	0.0375
Bit Diffusion (one-hot encoding)	0.0395
Bit Diffusion (bit-encoding)	0.0414

## Footnotes

1 Code available at https://github.com/jzhoulab/ddsm

2 It is also known as the univariate Wright-Fisher diffusion
