# Supplementary material for: Dirichlet Diffusion Score Model for Biological Sequence Generation
Source: ArXiv. 2023 Jun 16:arXiv:2305.10699v2. Originally published 2023 May 18. Preprint. [Version 2] (PMC10246113)
Supplement: 1 [file NIHPP2305.10699V2-supplement-1.pdf]

A. Supplemental Information for Dirichlet Diffusion Score Model

A.1. Transition Density Function of Jacobi Diffusion Process

For Jacobi diffusion process

$$\mathrm{d}\mathbf{x} = \frac{s}{2}[a(1-\mathbf{x}) - b\mathbf{x}]\mathrm{d}t + \sqrt{s\mathbf{x}(1-\mathbf{x})}\mathrm{d}\mathbf{w},$$

the transition density function is

$$p_{a,b}(x^t|x^0) = \mathcal{B}_{a,b}\left(x^t\right)\sum_{n=0}^{\infty}\frac{e^{\lambda_n t}}{d_n}R_n^{(a,b)}\left(x^0\right)R_n^{(a,b)}\left(x^t\right) = \mathcal{B}_{a,b}\left(x^t\right)\left(1+\sum_{n=1}^{\infty}\frac{e^{\lambda_n t}}{d_n}R_n^{(a,b)}\left(x^0\right)R_n^{(a,b)}\left(x^t\right)\right),$$

where $\mathcal{B}_{a,b}(x_t)$ is the **Beta**(a,b) density,

$$R_n^{(a,b)}(x) = P_n^{(b-1,a-1)}(2x-1)$$

denotes the n-th order modified Jacobi polynomial of order n.

$$d_n = \frac{a_{(n)}b_{(n)}}{(a+b)_{(n-1)}(2n+a+b-1)n!}$$

is the n-th order constant.

$$a_{(n)} = \frac{\Gamma(a+n)}{\Gamma(a)} = \prod_{k=0}^{n-1}(a+k)$$

denotes rising factorial also known as the Pochhammer symbol and

$$P_n^{(\alpha,\beta)}(x) = \frac{\Gamma(\alpha+n+1}{n!\,\Gamma(\alpha+\beta+n+1)}\sum_{m=0}^{n}\binom{n}{m}\frac{\Gamma(\alpha+\beta+n+m+1)}{\Gamma(\alpha+m+1)}\left(\frac{x-1}{2}\right)^m$$

is the Jacobi polynomial. $R_n^{(a,b)}(x)$ are eigenfunctions of the generator of the Jacobi diffusion process (Steinrücken et al., 2013a; Griffiths & Spano‘, 2010). The corresponding eigenvalues are $\lambda_n = -\frac{1}{2}sn(n-1+a+b)$.

A.2. Choosing Speed Factor s in Jacobi Diffusion Processes for Dirichlet Diffusion Score Model

Let us recall Jacobi diffusion process from Section 2.2:

$$\mathrm{d}\mathbf{x} = \frac{s}{2}[a(1-\mathbf{x}) - b\mathbf{x}]\mathrm{d}t + \sqrt{s\mathbf{x}(1-\mathbf{x})}\mathrm{d}\mathbf{w}.$$

The choice of s affects the convergence speed of Jacobi diffusion process while having no effect on the stationary distribution. In other words, the diffusion process converges to stationary distribution faster with higher s.

For modeling 2-category data with univariate Jacobi diffusion processes, the choice of s only affects the appropriate selection of maximum time in the diffusion model, which should be chosen inversely proportional to s. For modeling k-category data with multivariate Jacobi diffusion process by stick-breaking construction, the selection of s can affect the relative convergence speed for each independent univariate Jacobi forward diffusion processes.

We propose two options of s:

(i) The first option, $s = 1$, was empirically observed to achieve uniform convergence speed in $\mathbf{x}$ space across dimensions corresponding to different categories. This also generates samples that are nearly identical to fast sampling strategies described in Section A.4. Choosing $s = 1$ generally requires selecting a lower maximum time for more categories, to compensate for the faster convergence with higher k.

(ii) The second option, $s = \frac{2}{a+b}$, ensures uniform converge speed in the $\mathbf{v}$ space. This is motivated by choosing the first eigenvalue of the transition density function $\lambda_1 = -\frac{1}{2}s(a+b)$ to be equal across Jacobi diffusion processes. It also allows conveniently keeping a fixed maximum time for diffusion modeling (e.g. 4), regardless of the number of categories k in the data or the a, b parameters of the Jacobi diffusion.

We tested both variants of s and obtained good empirical results. Hence, these choices are often interchangeable. We used $s = 1$ for Sudoku generation and $s = \frac{2}{a+b}$ for promoter sequence design. The binarized MNIST application uses only the univariate Jacobi diffusion process where the two options are equal. We recommend comparing the choices on specific applications.

A.3. Weighting Function for Score Matching Loss Invariant to Change-of-Variable

Here we will show that the proposed weighted score matching loss is invariant to change-of-variable for any SDE. To first show that the unweighted score matching loss is not invariant to change-of-variable, we consider change of variable by any bijective, differentiable function $\mathbf{x} = h(\mathbf{v})$. Applying the change-of-variable equation for probability density function, the unweighted score matching loss

$$\left\| \frac{\partial \log p_{\mathbf{x}}(\mathbf{x})}{\partial \mathbf{x}} - \frac{\partial \log q_{\mathbf{x}}(\mathbf{x})}{\partial \mathbf{x}} \right\|_2^2 = \left\| \left(\frac{\partial \log p_{\mathbf{v}}(\mathbf{v})}{\partial \mathbf{v}} - \frac{\partial \log q_{\mathbf{v}}(\mathbf{v})}{\partial \mathbf{v}} \right) \frac{\partial \mathbf{v}}{\partial \mathbf{x}} \right\|_2^2$$

is not invariant to the change of variable due to the extra $\frac{\partial \mathbf{v}}{\partial \mathbf{x}}$ term. We now show that the loss function Equation 8 (also shown below) is invariant to change of variable $\mathbf{x} = h(\mathbf{v}, t)$ (where $\mathbf{x} = h(\mathbf{v})$ is a special case).

$$L(\mathbf{v}, t) = \left\| \frac{\partial \log p_{\mathbf{v}}(\mathbf{v})}{\partial \mathbf{v}} - \frac{\partial \log q_{\mathbf{v}}(\mathbf{v})}{\partial \mathbf{v}} \right\|_{\mathbf{G}\mathbf{G}^T}^2 = \left(\frac{\partial \log p_{\mathbf{v}}(\mathbf{v})}{\partial \mathbf{v}} - \frac{\partial \log q_{\mathbf{v}}(\mathbf{v})}{\partial \mathbf{v}} \right) \mathbf{G}(\mathbf{v}, t)\mathbf{G}(\mathbf{v}, t)^T \left(\frac{\partial \log p_{\mathbf{v}}(\mathbf{v})}{\partial \mathbf{v}} - \frac{\partial \log q_{\mathbf{v}}(\mathbf{v})}{\partial \mathbf{v}} \right)^T$$

For Ito diffusion process

$$\mathrm{d}\mathbf{v} = \mathbf{f}(\mathbf{v}, t)\mathrm{d}t + \mathbf{G}(\mathbf{v}, t)\mathrm{d}\mathbf{w},$$

change-of-variable to $\mathbf{v}$ gives the following Ito diffusion process due to Ito's lemma

$$\mathrm{d}\mathbf{x} = \left\{ \frac{\partial \mathbf{x}}{\partial t} + \frac{\partial \mathbf{x}}{\partial \mathbf{v}} \mathbf{f}(\mathbf{v}, t) + \frac{1}{2} \operatorname{Tr} \left[\mathbf{G}(\mathbf{v}, t)^T \left(\frac{\partial^2 \mathbf{x}}{\partial \mathbf{v}^2} \right) \mathbf{G}(\mathbf{v}, t) \right] \right\} \mathrm{d}t + \frac{\partial \mathbf{x}}{\partial \mathbf{v}} \mathbf{G}(\mathbf{v}, t)\mathrm{d}\mathbf{w}.$$

Thus, $\mathbf{G}(\mathbf{x}, t) = \frac{\partial \mathbf{x}}{\partial \mathbf{v}} \mathbf{G}(\mathbf{v}, t)$. Plugging this in equation 8, we see that $L(\mathbf{x}, t) = L(\mathbf{v}, t)$.

A.4. Improving Sampling Efficiency for Data with High Number of Categories

The sampling strategy for the forward diffusion process presented in Section 3.3 requires drawing samples from $k - 1$ Jacobi diffusion processes for k-category data, which can be demanding when k is high. Here we describe a strategy to accelerate sampling, needing to effectively sample from only one univariate Jacobi diffusion process.

We assume that the stationary distribution is the flat Dirichlet distribution $\mathbf{Dir}(1, 1, \ldots, 1)$. We reorder the sequence of stick-breaking construction, starting from the dimension with value 1 in one-hot encoding first, which allows us to initialize with $v_1 = 1$ and diffuse with Jacobi diffusion $(a = 1, b = k - 1)$. This reordering allows all other v_i values to be drawn without sampling from Jacobi diffusion as they can be drawn directly from their stationary distribution $v_i \sim \mathbf{Beta}(1, k - i)$. The samples will be converted to x space and reordered back to the original order. Reordering does not change the initial or stationary distribution of the diffusion process.

This sampling strategy leads to exactly $k - 1$ fold speed up and lower memory consumption during precomputation of the samples and scores. During training time, we also observed it to be faster than the regular sampling (for example, 2.08 vs 2.87 ms for the fast sampling method vs regular sampling method for $10,000$ dimensions).

Empirically, the fast sampling method generates nearly identical samples as the original multivariate Jacobi diffusion process by stick-breaking construction with all s factors set to constant. We find it hard to detect any noticeable decrease in sample quality or in log likelihood, showing that the effect is likely very small (on a synthetic data set, we observed 2.08 bits /dim using the model trained with the fast sampling strategy, whereas the ground truth optimal likelihood is 2 bits/dim). For example, our Sudoku model is trained with the fast sampling strategy and achieves perfect accuracy in Sudoku generation (see Appendix C.2 for more details).

A.5. Improving Sample Quality by Biasing Reverse Diffusion Toward High-Density Areas

Compared to unbiasedly sampling from the learned model distribution, it is often desirable to sample near the high probability density regions. These regions often correspond to higher-quality samples. We propose a simple technique applied to reverse diffusion sampling without modifying the score model when a flat distribution is the stationary distribution (e.g., the flat Dirichlet distribution).

There are two equivalent modifications of the reverse diffusion process during sampling:

(i) increasing the maximum time of reverse diffusion by a factor of k while querying the score model (and diffusion coefficient if it is time-dependent) with time proportionally scaled back to the original; or

(ii) accelerating the reverse SDE by a factor c while keeping the maximum time and score model of reverse diffusion unchanged. More specifically, we have

$$\mathrm{d}\mathbf{x} = c\Big\{\mathbf{f}(\mathbf{x},t) - \nabla\cdot\big[\mathbf{G}(\mathbf{x},t)\mathbf{G}(\mathbf{x},t)^\top\big] - \mathbf{G}(\mathbf{x},t)\mathbf{G}(\mathbf{x},t)^\top\nabla_{\mathbf{x}}\log p_t(\mathbf{x})\Big\}\mathrm{d}t + \sqrt{c}\mathbf{G}(\mathbf{x},t)\mathrm{d}\overline{\mathbf{w}}.$$

We call these techniques *time dilation*.

The modified SDE can be considered a reverse-time SDE for the forward diffusion accelerated by c. However, the score function is biased upward since it is estimated from the original forward diffusion which converges to stationary distribution slower. Thus, the samples tend to lie in higher-density areas. Since time dilation drives samples toward high-density areas, sometimes it is only desired to do locally instead of globally. Therefore, we find it effective to achieve so by starting time-dilation only at later stages of reverse diffusion.

It is also important to note that time dilation does not increase the time complexity of the sampling unless the number of sampling steps is increased. For example, 128x time dilation on 3200 time steps involves only 3200 evaluations. We generally recommend increasing the number of sampling steps proportional to the time dilation.

Overall, time dilation represents a general technique that can be applied to any continuous-time diffusion model.

B. Implementation Notes of Dirichlet Diffusion Score Model (continued)

B.1. Implementation of the Jacobi Diffusion Transition Density Function

We compute Jacobi polynomials using dynamic programming. Hence, the time complexity of the algorithm is linear with respect to the number of terms in the Jacobi diffusion process. Table 4 provides information about the computation time of the Jacobi diffusion density function with up to 1000 terms in eigendecomposition for the input of 10000 dimensions.

Order of Jacobi Polynomials	10	50	100	500	1000
Runtime - Pytorch (ms)	0.27	1.33	2.33	9.33	19.8

Table 4. Runtime of Jacobi diffusion density function computation on PyTorch 1.10.1.

By choosing the number of terms for computing the Jacobi diffusion density function, one seeks a trade-off between running time and numerical issues. Table 5 contains the relative error for the log gradient of the Jacobi diffusion density function ($a = 1$, $b = 3$) for different time points, averaged over 1000 samples for each time point, using scores evaluated with 10000 terms as the ground truth. Table 5 shows that 1000 terms are sufficient for precisely computing the score for $t = 0.001$, 100 terms are sufficient for $t = 0.01$, and 20 terms are sufficient for $t = 0.1$.

We decided to be conservative and chose to use 1000 terms for all our experiments. For numerical accuracy, we recommend evaluating Jacobi diffusion with eigendecomposition up to the 1000th term as well, with double precision floating point arithmetic, for time greater or equal to 0.001. It is also recommended to perform the stick-breaking transform and its inverse transform in double precision. Using double precision in these steps generally incurs negligible performance costs and noticeably improves numerical accuracy.

As described in the main text, we can presample a dictionary of diffused samples for each independent Jacobi diffusion process at uniformly spaced time points to allow efficient training. In the next section, we will discuss it in more detail.

# of terms	t=0.001	t=0.01	t=0.1	t=1
10	11.2	14.0	1.1	0
20	10.8	7.4	0	0
50	7.5	0	0	0
100	0.2	0	0	0
200	0	0	0	0
500	0	0	0	0
1000	0	0	0	0

Table 5. The relative error for the log gradient of Jacobi diffusion density function for different time points, averaged over 1000 samples for each time point.

B.2. Efficient Sampling and Score Computation from Jacobi Diffusion Processes

The computational complexity of both sampling and score computation from diffusion process for k category data is $O(n)$, where n is the dimensions of the input (e.g., a length of 1000 sequence with 4 categories has 4000 dimensions). We note that our diffusion process is specifically designed to be linear with respect to the number of categories, whereas other choices of multivariate diffusion processes with Dirichlet stationary distribution may have quadratic complexity (e.g. multivariate Wright-Fisher diffusion) with respect to the number of categories.

While our diffusion evaluation is more expensive than the commonly used diffusion process with Gaussian stationary distribution, all Jacobi diffusion-related computations can be precomputed prior to training and do not add to training time. This is feasible because we only need to generate samples from two starting points, 0 and 1, for any categorical data.

Thus, we can presample a dictionary of diffused samples for each independent Jacobi diffusion process at uniformly spaced time points to allow efficient training. For most applications, it suffices to sample a dictionary containing $100,000$ diffusion samples for each of 400 uniformly spaced time points for each Jacobi diffusion process. The presampled dictionary can be saved and reused for applications using the same forward diffusion processes. This approach applies to not just the standard sampling approach but also the fast sampling approach in Section A.4.

Sampling during training is done by choosing randomly from the pre-sampled samples and scores. The sampling time is negligible compared to neural network training. Thus, training/sampling/likelihood evaluation processes have the same complexity as the previous score-based SDE diffusion model. For example, only 2.87ms is needed for both generating a sample and its score for $10,000$ dimensions combined.

We believe that the whole process can be further optimized since only time points very close to zero would require a high number of terms to ensure numerical accuracy (see Appendix B.1). These optimizations together with JIT-based speed up may eliminate the need for precomputation without slowing down the training, sampling, and likelihood computation processes.

B.3. Importance Sampling of Time During Denoising Score-Matching Training

Importance sampling is often needed to stabilize the training of diffusion model with likelihood weighting (Song et al., 2021), since the scores for the forward diffusion transition density functions are usually large when time is small. Importance sampling is thus used as a variance reduction technique that samples time non-uniformly during training. By sampling time points where the scale of the score is higher more often and down-weight the sample loss accordingly, the variance of the gradient can be reduced. We determine the importance sampling weight based on the scale of the scores at each time point observed empirically. While different choices of importance sampling weights can be used, we found

$$w(t) \propto \mathbb{E}_{p_0(\mathbf{v}^0)p(\mathbf{v}^t|\mathbf{v}^0)} \left\| s\mathbf{v}(1-\mathbf{v})\nabla_{\mathbf{x}} \log p\left(\mathbf{x}^t \mid \mathbf{x}^0\right) \frac{\partial \mathbf{x}}{\partial \mathbf{v}} \right\|_F ,$$

where the norm is the Frobenius norm, to be a good choice for most applications.

B.4. Score Model Design

While the architecture of the score model should be designed for the specific data type and problem. There are several aspects of score model design that are shared in common.

First, the score model should take continuous time t as input, we found Gaussian Fourier projection to work well as the time embedding function following Song et al. 2020 which in turn followed Tancik et al. 2020.

Second, as discussed in Section B, the scale of the score function is dependent on time, especially when t is small. We can leave it to the score model to learn this time dependency or introduce a time-dependent scaling explicitly in the score model thus the model needs to learn the residual dependencies on time and input. For example, the last layer output can be multiplied with the time-dependent weight $w(t)$ in the above section with linear interpolation between time points.

B.5. Sampling

We used Euler Maruyama sampler and the modified version with time dilation (Section A.4) for all our applications for simplicity. Many improved sampling approaches have been proposed for more efficient sampling. We leave the exploration of applying these sampling approaches with Dirichlet diffusion score models for future research.

We discretize the samples by using argmax to choose the sampled category among k categories, even though mapping samples to discrete samples is trivial for trained models since the samples are generally close to 1 in one category and close to 0 in all other categories.

B.6. Selection of the Minimum Time for Diffusion Processes

The score of the transition density function at $t = 0$ does not exist and the score at very small t tends to become very large and cause numerical issues. Thus in practice, a choice of the minimum time used for training, sampling, and likelihood or ELBO evaluation is needed. With typical choices for diffusion parameters suggested in the manuscript, a minimum time of 0.01 or 0.001 is sufficient.

B.7. Randomization of Stick-Breaking Construction Order

We do not in general observe the order of stick-breaking construction in multivariate Jacobi diffusion to affect the model performance or samples. However, it is possible to enforce order invariance by randomizing the stick-breaking construction order during training or sampling. As the score model is formulated in $\mathbf{x}$ space, it can be converted to $\mathbf{v}$ space with any stick-breaking transform order.

B.8. The empirical assessment on the tightness of ELBO

For measuring the gap, we created a simple test case with 4 categories for which the ground truth data density is known. Table 6 contains the measured gap between the ELBO and the ground truth likelihood. It represents an upper bound of the ELBO variational gap.

$t_{\tilde{0}}$	Gap (bits/dim)
0.001	0.0023
0.002	0.0045
0.005	0.0111
0.010	0.0219

Table 6. Empirical assessment of the gap between our ELBO and the ground truth likelihood. $t_{\tilde{0}}$ is a time close to 0.

We conclude that this gap should be tight enough for most applications and can be further tightened by lowering $t_{\tilde{0}}$, where $t_{\tilde{0}}$ is a time close to 0.

B.9. Comparison of Time Dilation Approach with Other Improving Sample Techniques

We compared the proposed time dilation approach with predictor-correct sampling using the sudoku generation task (see Appendix C.2 for more details about the sudoku experiments). Table C.2 contains results from the Predictor-Corrector sampler with the number of corrector steps being 1, 3, 7 and from the time dilation approach. Both techniques use the same number of evaluations. From Table C.2, we conclude that time dilation approach consistently shows better performance. However, it is important to note that time dilation is a *biased* sampling technique (i.e., sample preferentially from high-density areas), whereas predictor-corrector sampling is intended for unbiased sampling.

Model	Accuracy
**Time dilation 8x**	**100**
Time dilation 4x	99.88 ± 0.06
Time dilation 2x	98.87 ± 0.16
Predictor-Corrector (7 corrector steps)	98.86 ± 0.26
Predictor-Corrector (3 corrector steps)	97.70 ± 0.26
Predictor-Corrector (1 corrector step)	95.16 ± 0.47
Baseline	95.08 ± 0.46

Table 7. Accuracy comparison of different sampling methods.

C. Application Details

In this section, we provide more details about the experiments and applications.

C.1. Binarized MNIST

The model architecture is adopted from Ho et al. 2020, and replaces the time embedding with Gaussian Fourier projection-based continuous-time embedding. The model is trained with univariate Jacobi diffusion with $s = 1$, time-dependent weight-based scaling in the score model (Section B.4), minimum time of 0.001, and maximum time of 4.

C.2. Sudoku Generation and Solving

The Sudoku training samples are fully-filled Sudokus sampled from the Sudoku generation code (`https://github.com/Kyubyong/sudoku/blob/master/generate_sudoku.py`, which is itself an adaptation of Sudoku generation code from `https://www.ocf.berkeley.edu/~arel/sudoku/main.html`). Sudoku puzzles are randomly generated puzzles by the "pluck" method of this code. We also measured the success rate of this Sudoku generation algorithm, which iteratively fills the Sudoku puzzle with numbers with no conflict, until it is no longer possible. As a baseline, the heuristic algorithm only has 0.31% accuracy.

The Sudoku transformer is a 20-block transformer architecture with the attention-bias style relative positional embedding, as described in the main text. The Sudoku transformer model is trained with the fast sampling strategy described in Section A.4 with maximum time 1.

For generation and solving Sudoku puzzles, we used Euler Maruyama sampler with and without time-dilation technique for reverse diffusion sampling. $100k$ steps where k is the time-dilation factor are used. To estimate the accuracy of easy and hard Sudoku puzzles with a single sample and 128x time-dilation, we used 3200 steps. To solve Sudoku with multiple samples, 8x time dilation with 200 steps was used, and we keep generating new samples until the generated sample solved the Sudoku puzzle.

C.3. Sudoku performance comparison with baseline methods

Table 8. Sudoku generation and solving accuracies for *single samples*, in comparison with baseline diffusion methods. We trained all models with the Sudoku transformer architecture.

Task	Method	Accuracy (%)
Generation	**DDSM (time dilation 8x)**	**100**
	DDSM (time dilation 4x)	99.88 ± 0.06
	DDSM (time dilation 2x)	98.87 ± 0.16
	DDSM (time dilation 1x)	95.08 ± 0.46
	Bit Diffusion	99.60 ± 0.11
	D3PM-uniform / Multinomial Diffusion	98.90 ± 0.18
Solving	**DDSM (time dilation 8x)**	**98.26 ± 0.18**
	DDSM (time dilation 4x)	97.54 ± 0.18
	DDSM (time dilation 2x)	96.45 ± 0.32
	DDSM (time dilation 1x)	93.85 ± 0.42
	Bit Diffusion	7.48 ± 0.55
	D3PM-uniform / Multinomial Diffusion	7.37 ± 0.58

C.4. Human Promoter Sequence Design

You can refer to Appendix F.1 if you are looking for more background information on the promoter design problem.

For the preparation of the dataset, we first obtained human TSS position annotation from the FANTOM-CAT catalog using the Level 3 (Robust) annotations. We obtained transcription initiation signal profiles measured by CAGE from the FANTOM project (Consortium et al., 2014). FANTOM CAGE datasets were downloaded from `https://fantom.gsc.riken.jp/5/datafiles/latest/`. We averaged all CAGE profiles after applying $\log(x + 1)$ transformation to obtain a robust genome-wide transcription initiation signal profile.

The human genome sequences are retrieved from hg38, with each sequence 1024 bp centered at the annotated TSS position. The sequences are retrieved on the same strand as the annotated direction of transcription of the promoter. In total, 100,000 promoters with the highest expression are retrieved. The promoters are further split into the training, validation, and test sets based on chromosomes (chr8 and 9 for the test set, chr10 for the validation set, and all other chromosomes for the training set). In the training set, we also introduce the same amount of random shift of up to $+/-100$bp to the sequence and transcription initiation profile simultaneously. The sequences and transcription profile profiles at centered at a location within $+/-100$bp distance to the annotated TSS in this case. Random shifts are only used during training as a data augmentation and regularization technique.

The Promoter Designer model has a custom-designed 1D convolutional architecture. This conditional generation model concatenates the 4-dimensional $\mathbf{x}$ input and the 1-dimensional transcription initiation signal (CAGE) profile. The training uses $s = \frac{2}{a+b}$ Jacobi diffusion processes with maximum time 4. For sampling from the trained model, we used Euler Maruyama sampler with 100 steps.

For analysis of generated sequences and comparison with human genome sequences, we generated sequences conditioned on the test set transcription initiation profiles among the top 40,000 promoters. 5 sequences are generated for each transcription initiation profile. The human genome sequences for these test set promoters are used for comparison. For motif position distribution analysis, we used the following motifs from JASPAR database (Castro-Mondragon et al., 2022): TATA-box, AC0057:TBP/ZNF:TBP; GC-box, AC0524:KLF/SP:C2H2_ZF; CCAAT-box, AC0240:NFYA/NFYB:CBF/NF-Y. Sei model (Chen et al., 2022a) is trained with the same validation and test holdout chromosomes as Promoter Designer, and is thus ideal for evaluation of Promoter Designer. For Sei model prediction, we padded the input sequence to 4096bp with 0.25 and averaged the prediction for all H3K4me3 targets to obtain the predicted sequence promoter activity score.

For comparison with baseline diffusion model methods, we trained all models with the same Promoter Designer architecture, including retraining the DDSM model, using the same early stopping criterion (SP-MSE on the validation set).

D. Binarized MNIST Generation Examples Including Time-Dilation Experiments

We show below samples from the Dirichlet diffusion score model, with and without applying time-dilation (Appendix B.3) in sampling.

Consistent with our expectation that time-dilation produces samples biased toward high-density regions, we notice that samples with time-dilation generated more stereotypical digits. Further increasing time dilation also causes the samples to contain more “1”s, which is likely due to that “1” is slightly more common than other digits in the binarized MNIST dataset. The second most common digit “7” is also over-represented in highly time-dilation samples, consistent with the hypothesis that the non-uniform distribution among digits in the dataset is exaggerated by time dilation.

We get the best of both worlds i.e. increased digit quality while not biased toward overrepresented digits, but starting the time-dilation only at a later stage of reverse diffusion.

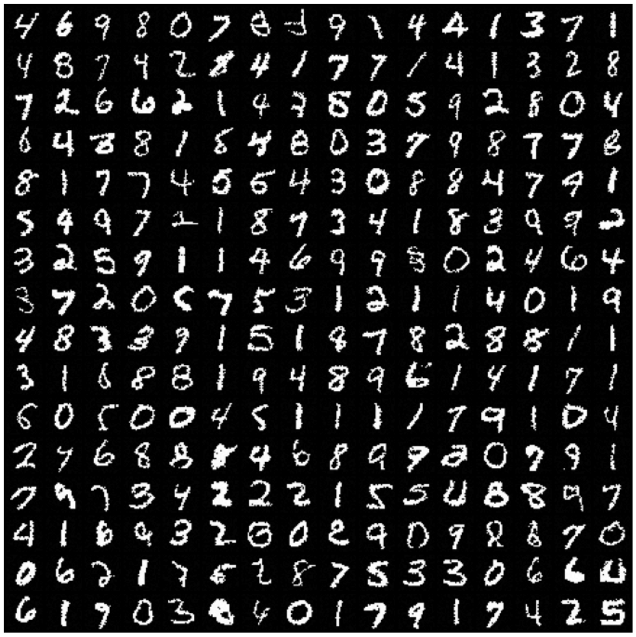

Figure D.5. Binarized MNIST samples from Dirichlet diffusion score model. Euler Maruyama Sampler with 100 steps.

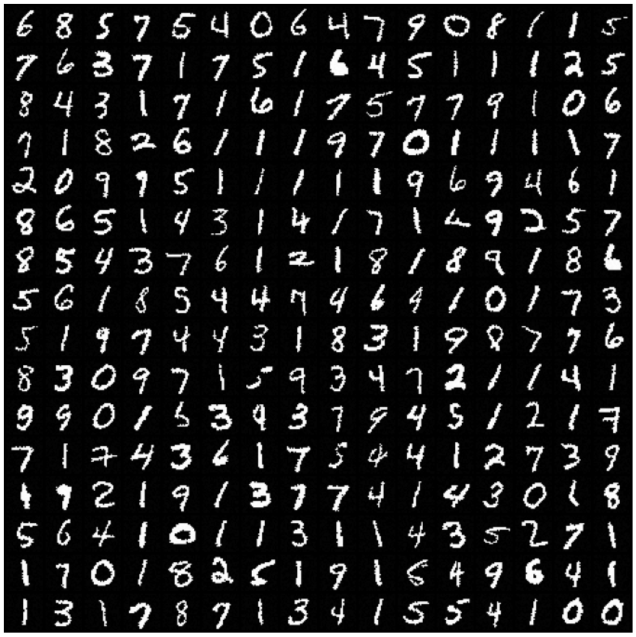

Figure D.6. Binarized MNIST samples from Dirichlet diffusion score model (2x time-dilation). Euler Maruyama Sampler with 2x time-dilation and 200 steps.

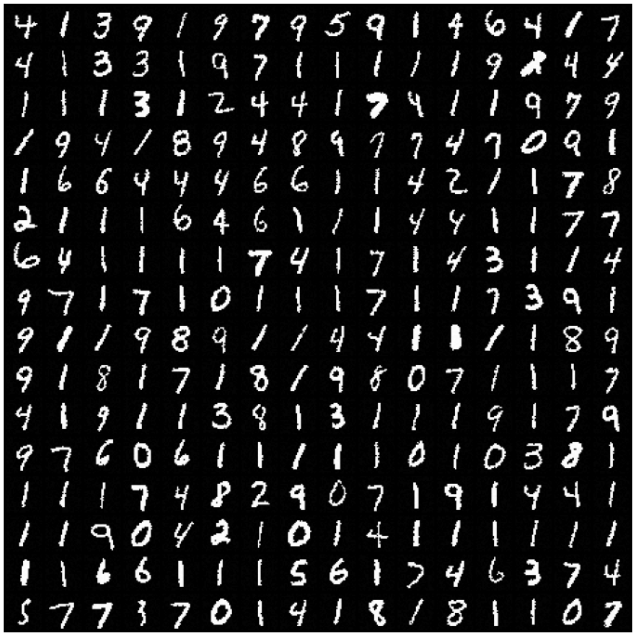

Figure D.7. Binarized MNIST samples from Dirichlet diffusion score model (4x time-dilation). Euler Maruyama Sampler with 4x time-dilation and 400 steps.

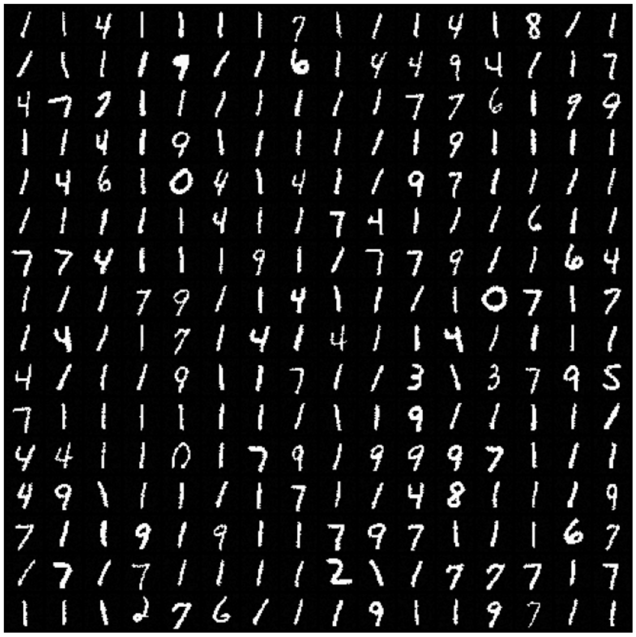

Figure D.8. Binarized MNIST samples from Dirichlet diffusion score model (8x time-dilation). Euler Maruyama Sampler with 8x time-dilation and 800 steps.

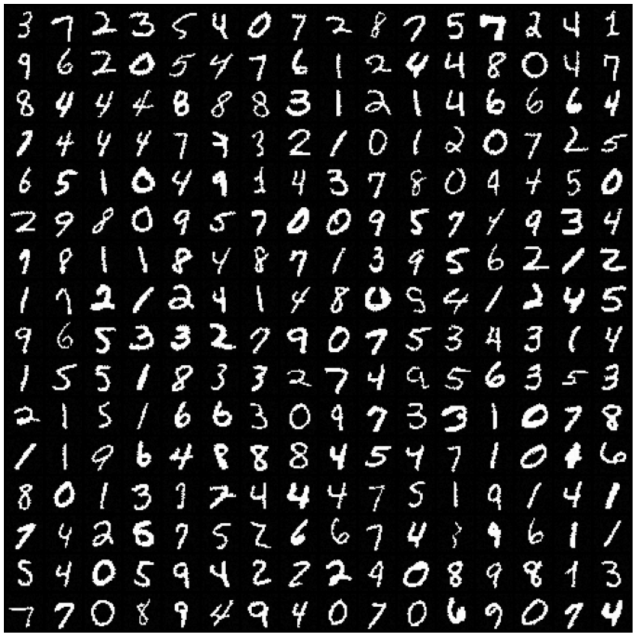

Figure D.9. Binarized MNIST samples from Dirichlet diffusion score model (8x time-dilation, time-dilation start time 25%). Euler Maruyama Sampler with 8x time-dilation started at 25% time point, with a total of 275 steps.

E. Sudoku Transformer Model Performance on the Hard Sudoku Dataset

The Sudoku transformer model solved all puzzles in an easy Sudoku dataset with 36 clues on average (Wang et al., 2019) and a hard Sudoku dataset with minimally 17 clues (Palm et al., 2018) when multiple samples are used. We also note that the current state-of-the-art results for supervised models are Recurrent Relational Network (Palm et al., 2018), which solves 96.7% of the 17-clue Sudoku puzzles within the hard dataset, and SATNET(Wang et al., 2019) which solved 98.3% of the easy dataset. Our method solved 100% puzzles, and this requires usually only one or two samples (mean = 1.19 samples) on the easy dataset and a high number of samples (mean = 753 samples) on the hard dataset. A single sample from our model solves 99.4% of the easy dataset and 42.4% of the hard dataset with 128x time-dilation. Recurrent Relational Network still have better accuracy when only a single sample is allowed for our model. However, our model is never trained on the Sudoku dataset or even in a supervised manner, as it was only trained on generating fully-filled Sudoku from a random Sudoku generator. Thus, these results are not directly comparable. Our result is the first in generating modeling of Sudoku to our knowledge, which already showed very strong performance and even surpass state-of-the-art approaches with supervised training with multiple samples are allowed.

On the hard dataset, we demonstrated the scaling of the number of samples required with the number of clues given (17 is the minimally possible number for Sudoku) (Figure E). We note that we are not optimizing this experiment to minimize the number of samples drawn but the overall time spent solving the puzzle, as we can significantly increase single-sample accuracy by applying more time dilation, at the cost of more computation per sample.

This task can serve well as a benchmark for strongly constrained discrete data generation or efficient reverse diffusion sampling methods. With our current setup, each sample is generated with 8x time-dilation and 200 steps, which is certainly not optimized given our use of a simple Euler Maruyama sampler modified to support time-dilation.

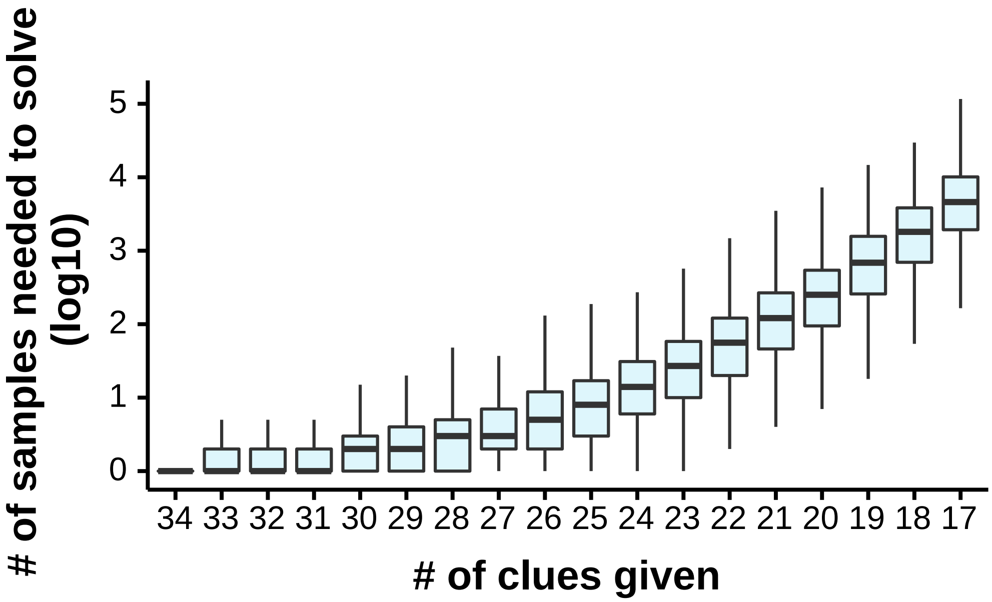

Figure E.1. Number of samples required to solve hard Sudoku puzzles by the number of clues.

F. Supplementary Information for Promoter Sequence Design

F.1. An introduction of the promoter sequence design problem and our study design without assuming prior knowledge of biology

DNA sequence is composed of 4 bases, or 4 nucleotides: A, C, G, T. The length unit of DNA sequence is basepair (bp) because DNA is usually double-stranded, and each base is paired with its complementary base on the other strand. The DNA base pairing rules are that 1) A and T are complementary to each other, 2) G and C are complementary, 3)the two strands go in opposite directions. Therefore, if a 10bp sequence reads CCAATTTAAG, and the other strand (its reverse complement) will read CTTAAATTGG following the base-pairing rule.

An important function of DNA sequence is to encode genes. For genes to function they have to be first transcribed to RNA (the DNA information is copied basepair-by-basepair to RNA molecules; while a cell has only two copies of the genome DNA, DNA can be transcribed to many RNA molecules). Promoters are sequences that determine where the transcription happens and partially determine how much transcription happens. The amount of transcription from a promoter can be called the "expression level" of a promoter. The starting point of transcription, or where DNA starts to be transcribed to RNA, is called the transcription initiation site or the transcription start site. Transcription can happen in many different positions within a promoter, but there is a single basepair that is annotated as the transcription start site (TSS) for every promoter in previously published annotations, which is usually but not always near where most of the transcription starts at.

Where and how much transcription happens can be measured by experimental methods such as CAGE (Cap analysis gene expression). From experimentally generated data we can obtain a transcriptional initiation signal profile, or more specifically how much transcription happens at every single basepair position, for every human gene promoter. A transcriptional initiation signal profile characterizes the transcriptional function of a promoter, including its expression level which can be obtained from the total amount of transcription over the entire promoter region.

If we have the ability to design promoter sequences that can produce any desired transcription initiation profile, we will also be able to finely control the expression level of any synthetic gene, such as genes producing bioengineering products like antibodies. We can also control very finely where the transcription starts, which may enable potential future applications. On the other hand, it can be a tool for improving our understanding of the mechanism of transcription initiation, or precisely how sequence drives transcription, which is not fully understood.

Therefore, the goal of the promoter sequence design task is to conditionally generate sequences that will produce the same transcription initiation signal profile as the conditional input. To our knowledge, no prior method exists for this task.

To evaluate how well the model works, we first compared the model-designed sequences with real human genome sequences behind transcription initiation signal profiles from the test set. We showed that they indeed have very similar properties, including the base (or nucleotide) composition at every position relative to the transcription start site, as well as the location-specific distribution of known promoter motifs. Known promoter motifs are sequences that are known to frequently appear at promoters and likely play a role in transcription initiation. The examples of the most common promoter motifs are TATA-box (TATAAAA), GC-box (GGGCGGG or CCCGCCC), CCAAT-box (CCAAT or ATTGG). Therefore, we showed that they also show up in our designed sequences and at similar locations with similar frequencies compared to the real genome sequences. We note these are not the only important sequences and our knowledge of sequence rules that drive transcription is still far from complete.

Finally, we used a deep learning model, Sei, that can predict promoter activity to evaluate whether our design sequences will produce the desired activity levels as given in the conditional input: the transcription initiation signal profile. We grouped the transcription initiation signal profiles into 10 groups based on the percentile of expression levels, from the lowest group (0-10%) to the highest group (90%-100%). Encouragingly, the predicted promoter activities of model-designed sequences closely resemble those of the human genome sequence corresponding to each expression group. This suggests that the model can generate both low-activity promoters and high-activity promoters by controlling the input transcription initiation signal profile.

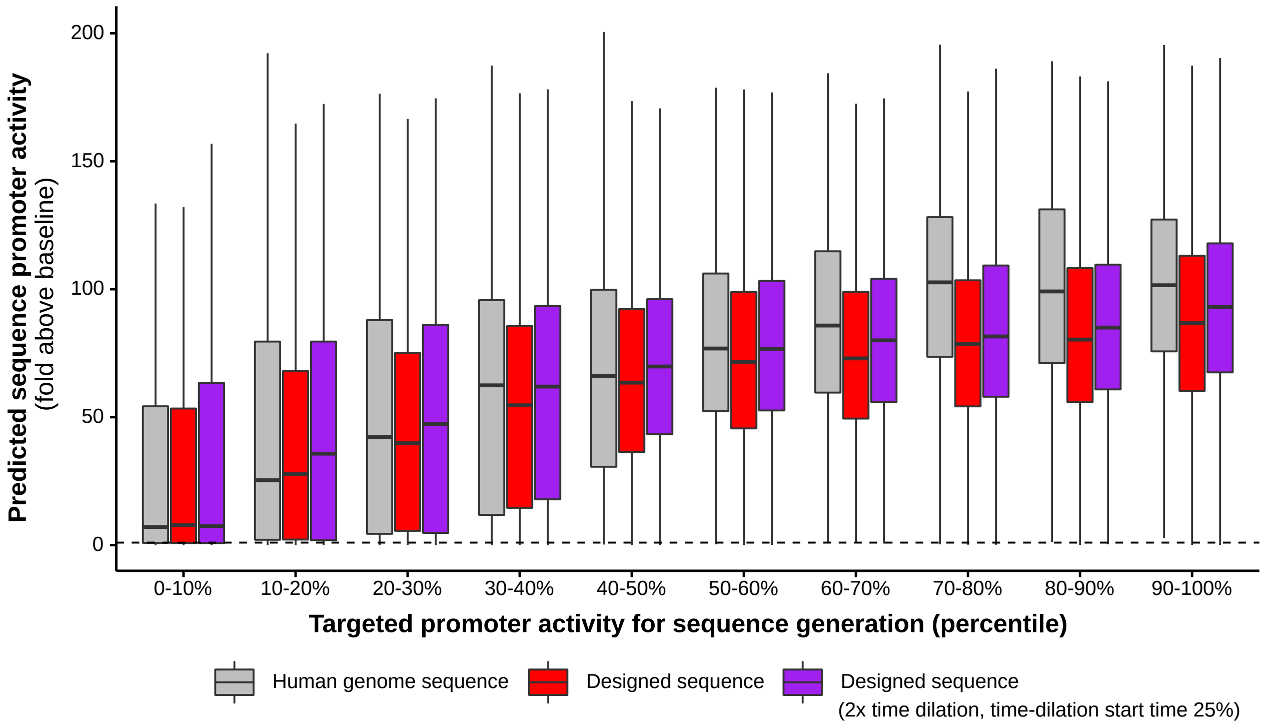

Figure F.1. Time-dilation (2x) slightly improves promoter activity predicted from sequence by Sei. Generated sequences are grouped by the targeted promoter activity level (x-axis). Y-axis shows predicted H3K4me4 probability (average across cell types), divided by baseline prediction for average genomic sequences.

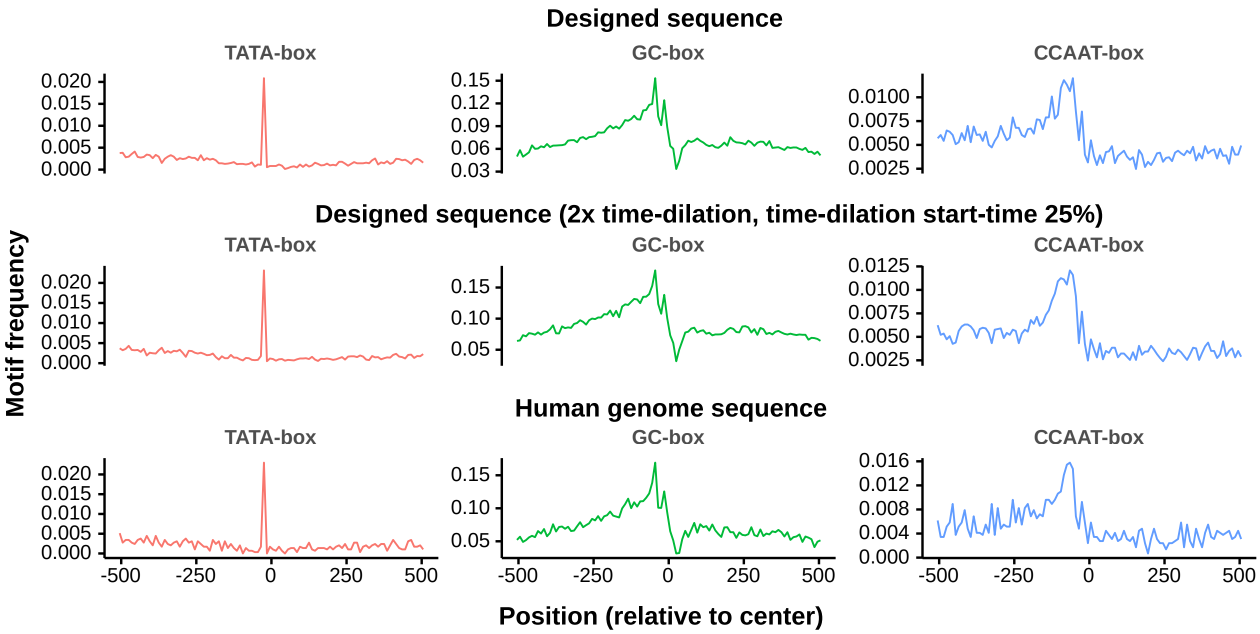

Figure F.2. Position-specific motif distribution of designed and generated sequences. The time-dilation increased the known motif frequencies.

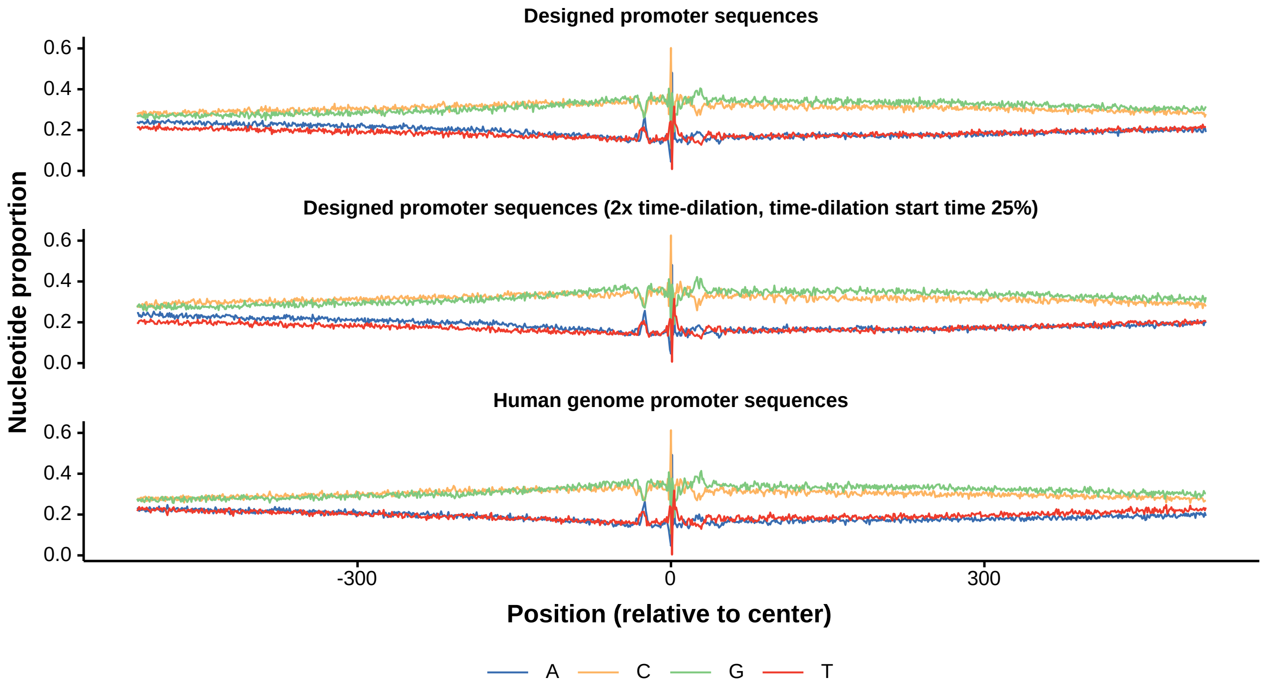

Figure F.3. Position-specific nucleotide composition of designed and generated sequences. The time-dilation leads to slightly more biased nucleotide composition.
